# Crystal orientation and crystal structure of paramagnetic α-Al under a pulsed electromagnetic field

**DOI:** 10.1038/s41598-020-67352-4

**Published:** 2020-06-30

**Authors:** Qingwei Bai, Jun Wang, Shuqing Xing, Yonglin Ma, Xinyu Bao

**Affiliations:** 1 Key Laboratory of Integrated Exploitation of Bayan-Obo Multi-Metallic Resources, Inner Mongolia University of Science and Technology, No. 7 Arding Street, Baotou, 014010 Inner Mongolia People’s Republic of China; 2 School of Material and Metallurgy, Inner Mongolia University of Science and Technology, No. 7 Arding Street, Baotou, 014010 Inner Mongolia People’s Republic of China; 3 Inner Mongolia Autonomous Regional Engineering Technology Research Center of Sustainable Exploitation of Rare Earth Secondary Resources, Inner Mongolia University of Science and Technology, Baotou, 014010 People’s Republic of China

**Keywords:** Materials science, Structural materials

## Abstract

The intermittent electromagnetic fields with a large $\partial \mathrm{B}/\partial \mathrm{t}$ can enhance the properties of ferromagnetic materials and significantly affect paramagnetic materials. In this study, the effect of a pulsed electromagnetic field on the crystal orientation of the primary phase and microstructure evolution of an Al–Zn–Mg–Cu alloy was investigated. A mathematical model was developed to describe crystal rotation under a pulsed electromagnetic field. The model predictions show that the magnetic energy difference generated by the magnetic anisotropy of the primary crystal produces primary phases with sizes of 225–100 μm to rotate into a <111> preferred orientation. The lattice constant, the interplanar spacing, and the microstrain increase with the duty cycle of the pulsed magnetic field, especially for the (111) and (200) crystal planes. This study provides preliminary theoretical support for using pulsed electromagnetic fields to control the orientation and microscopic properties of materials.

## Introduction

Some organic and inorganic materials exhibit unusual phenomena under electromagnetic fields. Grain rotation, deformation, and orientation changes are generated in metallic materials during electromagnetic solidification processing, and levitation and structural transformations are observed for nonferromagnetic materials. Thus, the magnetic force is considered to be the main factor in materials processing. The magnetic force has two components. The parallel component causes a magnet pulls ferromagnetic and paramagnetic materials and repels diamagnetic materials. The rotational component results in the generation of a Lorentz force from the interaction of the current and the external magnetic field. The parallel magnetic force is mainly used for magnetic separation, magnetic levitation^1^, and to measure the magnetic susceptibility^2^. The magnetocrystalline anisotropy of materials determines the magnetic susceptibilities in different crystal planes. Thus, the rotational magnetic force can be used to construct materials with ideal crystal orientations and texture distributions^3–8^. The magnetic anisotropy is widely used to control crystal orientation, for example, to realize magnetic directional alignments of an undoped Cu–O superconducting magnet and Zn film are achieved. For the FCC (face-centered-cubic) crystal, when the magnetic susceptibility along the direction parallel to the c-axis of the unit crystal (${\chi }_{c}$) is higher than that perpendicular to the c-axis (${\chi }_{\mathrm{a}\mathrm{b}}$), the unit crystal grows along the c-axis, which is parallel to the external field, H^9–11^. Magnetic anisotropy in ion-doped materials results from single-ion anisotropy in the magnetic field^12^.

Recently, Liu et al.^13^ investigated the influence of a magnetic field on the crystal orientation and magnetic properties of Fe-4.5 wt% Si. The crystal orientation of the Fe-4.5 wt% Si alloy was rotated into the <100> axis, and strong Goss texture was obtained under a 6-T high static magnetic field. Zhong et al.^14^ found that an Al-4.5 Cu free crystal rotated in the melt state to align with the <310> crystallographic axis in the direction of the magnetic field. Increasing the magnetic field intensity resulted in a random-<111>–<113>–<110> transformation of the (Tb, Dy) Fe_2_ orientation^15^. For crystal orientation applications, Asai et al.^16^ summarized the anisotropy values of several nonmagnetic materials after electroplating, vapor deposition, solidification, and heat treatment under a high magnetic field. Ferreira et al.^17^ mathematically modeled the crystal orientation during nucleation and growth processes under a high magnetic field. However, fundamental research studies have not been performed to further explain and predict these phenomena.

**Figure 1:**
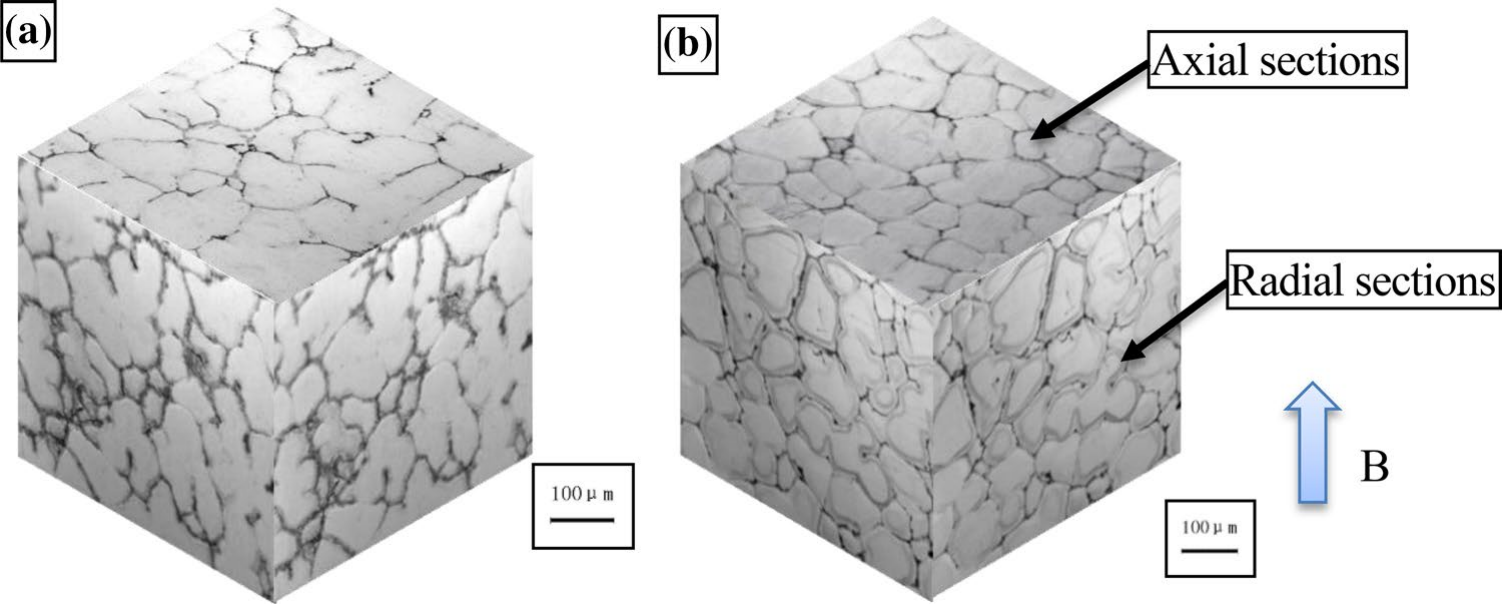
Central solidified structure of 7A04 aluminum: (**a**) untreated and (**b**) treated with 20% pulse duty cycles^20^. ©2017 ASM International.

**Figure 2:**
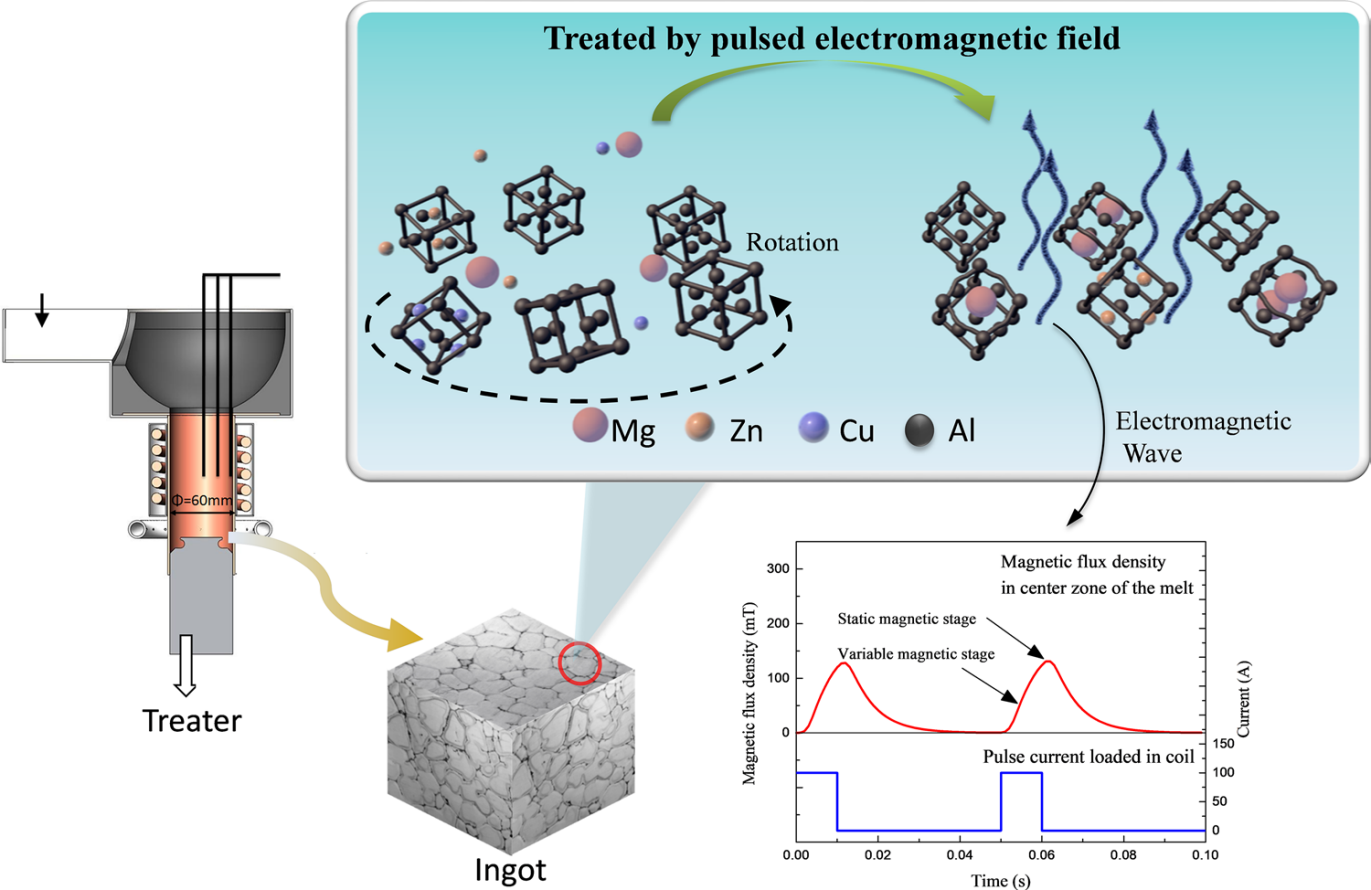
Crystal rotation and microstructure evolution under a pulsed electromagnetic field^20^. ©2017 ASM International.

Pulsed magnetic fields excite an intermittent noncontact electromagnetic field with a large $\partial \mathrm{B}/\partial \mathrm{t}$ that can significantly affect a solidified structure^18,19^. Compared with a steady-state DC magnetic field, the pulse width of a pulsed magnetic field is on the order of milliseconds or microseconds and can produce a high magnetic flux density. This pulsed electromagnetic effect can be used to prepare ferromagnetic, paramagnetic, and diamagnetic materials. Pulsed electromagnetic fields are convenient to use, have low energy consumption, and produce good structural improvements and are therefore one of the most promising techniques for material processing. We previously found^20–22^ that a pulsed electromagnetic field promoted the nucleation and solidified structural transformation of paramagnetic Al–Zn–Mg–Cu alloys. A rose structure with a coarse grain size was transformed into a fine and rounded spherical structure with improved strength and plasticity (see Fig. 1). A pulsed electromagnetic field decreases the critical Gibbs free energy for nucleation to occur in a system, which is an important factor in increasing the nucleation rate and refining grains. However, the mechanical properties of materials depend strongly on the crystal orientation and microstructure. Lorentz force causes melt motion, and it plays another role in melt at the same time. From Lenz’s law, the rotation of an electrically conductive substance in a magnetic field produces a Lorentz force from the interaction between the rotational motion and the ∂B⁄∂t generated to suppress particle rotation. The Lorentz force reduces the influence of particle displacement on the orientation. The distribution of crystal defects, such as vacancies, dislocations, and stacking or twin faults, can produce microstrain in the microstructure. A high dislocation density and significant microstrain in the crystal phase increases the total strength and plasticity of the crystal^23,24^. In this paper, a pulsed electromagnetic field was applied during the solidification of an Al–Zn–Mg–Cu alloy. The graphical abstracts about the crystal rotation and microstructure evolution under a pulsed electromagnetic field were exhibited in Fig. 2. The formation mechanism of the preferred crystal orientation under the Lorentz force was theoretically analyzed, and a mathematical model for the rotation of the primary phase under a pulsed magnetic field was constructed. A method was proposed for controlling the crystal anisotropy during solidification, and the evolution rule of the microstructure was determined in terms of the lattice constant, the microstrain, etc. This study provides a theoretically based mechanism by which pulsed electromagnetic field processing technology improves solidified structures.

## Experiment and process

**Figure 3:**
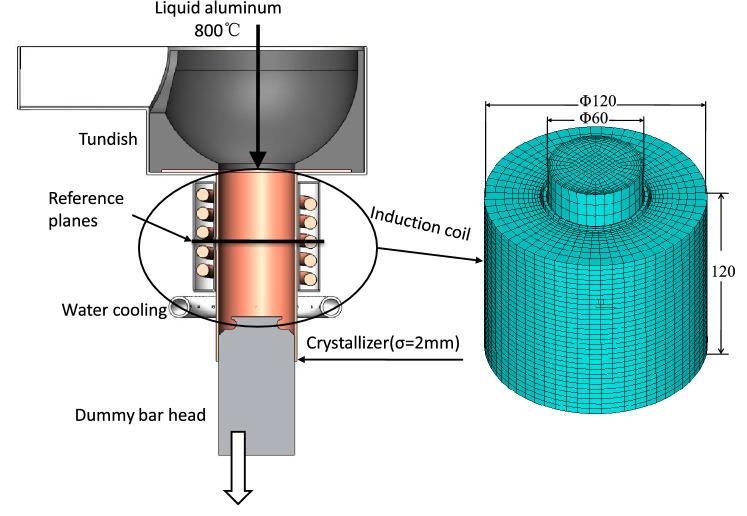
Schematic and 3D simulation model of an electromagnetic pulse caster^20^. ©2017 ASM International.

The test material was Al–Zn–Mg–Cu, a superhard aluminum alloy used in aerospace applications. The chemical composition of the alloy is listed in Table 1. This material has a wide solid–liquid phase region, a liquidus temperature of 654 °C, and a complete solidus temperature of 528 °C. Figure 3 shows the experimental device, which consists of a pulsed electromagnetic field generator (comprising a water-cooled induction coil and an iron core), a pulsed power supply, a resistance heater, a cooling system, an induction melting furnace, a crystallizer, and a tundish. The crystallizer was preheated to 500 °C by an internal resistance heater, and the alloy was melted in the medium-frequency induction melting furnace at 800 °C for 10 min. The resistance heater was then removed from the crystallizer. The melt was poured into the crystallizer (Φ = 60 mm) and subjected to pulsed electromagnetic treatment until the temperature dropped below 300 °C. The water cooling device was activated to rapidly cool the sample. The parameters of the electromagnetic pulse were as follows: a peak current of 200 A, a pulse frequency of 20 Hz, a magnetic flux density of 153 mT, and a pulse duty cycle (the time ratio between the pulse discharge and the total pulse period for each period) of 20% or 40%. The solidified specimens were analyzed using X-ray diffraction (XRD). A three-dimensional finite element method (3D-FEM) was employed to investigate solidification under the pulsed electromagnetic field. A mathematical-physical model, including the pulse coil, copper mold, and melt, was established. The Lorentz force was initially calculated using ANSYS EMAG and used as a source in ANSYS FLUENT to calculate the flow and temperature fields. The detailed governing equations and associated boundary conditions can be found in the literature^21^.

**Table 1:** Alloy chemical composition (wt%).

Si	Cu	Mg	Zn	Ti	Al
0.09	1.92	2.45	5.22	0.04	Bal

## Preferred crystal orientation

### Solidification environment under pulsed magnetic field

**Figure 4:**
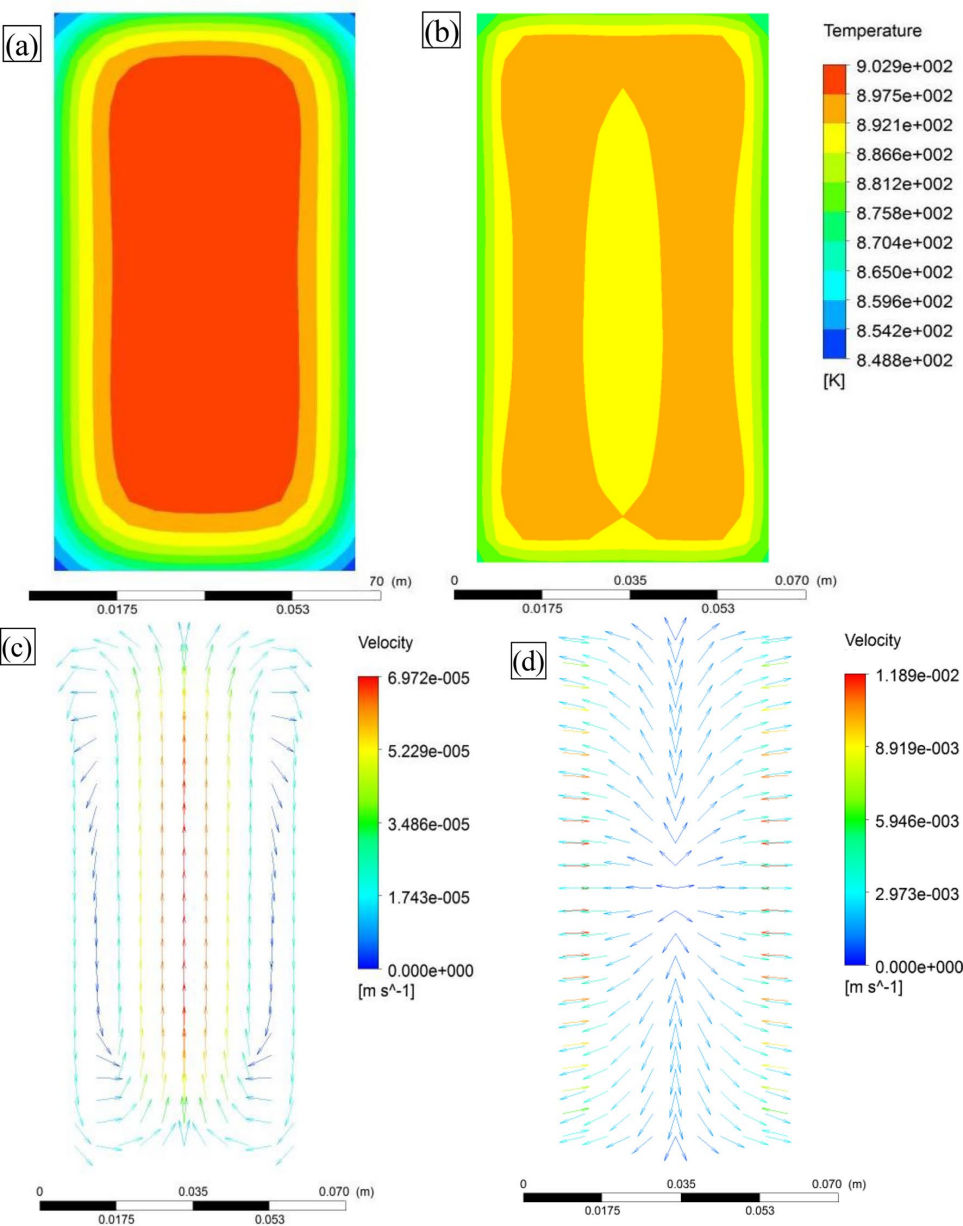
Temperature field (**a**) before and (**b**) after pulsed electromagnetic field application; and flow field (**c**) before and (**d**) after pulsed electromagnetic field application.

Figure 4 shows the transient numerical simulation results of the flow and temperature fields of the longitudinal melt section before and after applying the pulsed electromagnetic field. In the absence of the pulsed magnetic field, the heat flux is directed from the center to the edge of the melt, and two symmetric and opposite vortex rings form in the longitudinal section. The application of the pulsed electromagnetic field (initial temperature: 923 °C, duty cycle: 20%) induces forced convection in the melt, which decreases the temperature gradient. The primary phase nucleates and grows in the quasisteady environment. The eddy-current effect is weakened, which reduces the impact on analyzing the orientation process of the primary phase.

### Effect of pulsed electromagnetic field on crystal orientation

**Figure 5:**
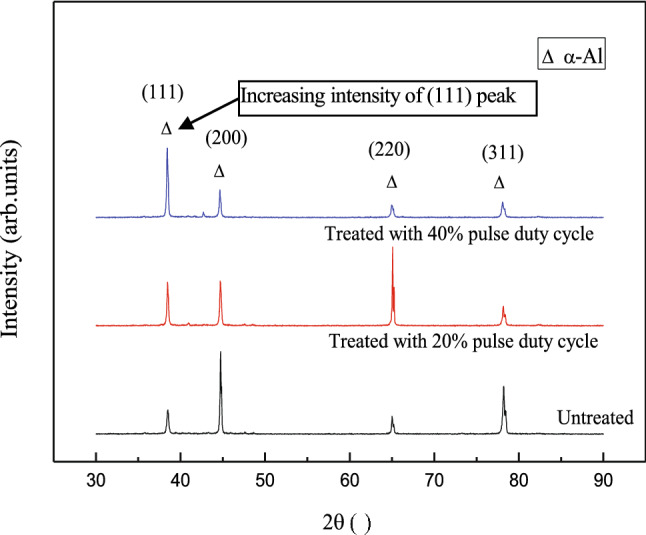
XRD of solidified Al–Zn–Mg–Cu alloy.

The shape and peak position of the XRD patterns showed that the pulsed electromagnetic field treatment changed the microstructure and several properties of the solidified alloy. The crystal structures of the Al–Zn–Mg–Cu alloy ingot cores were analyzed using a D8 ADVANCE XRD diffractometer. The scanned section was perpendicular to the direction of the magnetic field, and the analysis results are shown in Fig. 5. The diffraction peak corresponding to the (111) plane of α-Al crystals was enhanced by pulsed electromagnetic field treatment, indicating that applying the pulsed magnetic field in the axial direction of the specimen promoted atom packing in the preferred <111> orientation. However, the diffraction peak intensities corresponding to the (200) and (311) planes gradually decreased as the pulse duty cycle increased, and the magnetic field gradually suppressed crystal growth along these two orientations. Three conditions must be satisfied for a magnetic field to control grain orientation: (1) the atomic clusters in the system must overcome the energy barrier to form a critical nucleus with radius *r* *; (2) the magnetization energy must be higher than thermal fluctuations; and (3) the magnetic susceptibility in the <111> direction of the alloy must be greater than along the a-, b-, and c-axes, i.e., $E_{a, b, c} > E_{{\left( {111} \right)}}$, such that the magnetization energy difference causes the primary phase to rotate under the pulsed magnetic field, whereby the <111> orientation is rotated toward the magnetic field. Preferential crystal growth mainly depends on the atomic arrangement order. The close-packed (111) plane has the lowest surface energy and a compactness of up to 74%. Atoms always preferentially arrange along low-energy planes, which are easy to stabilize. Atoms are arranged relatively loosely on the (200) plane with a lower coordination number, which makes a crystal grow slowly along the <h00> direction.

**Figure 6:**
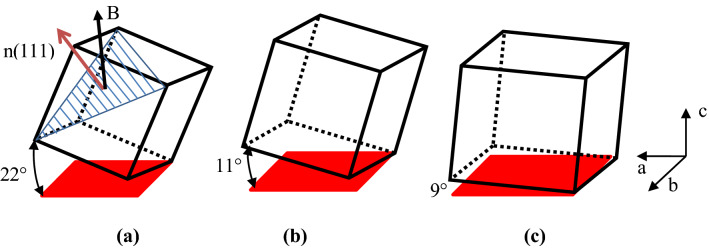
Schematic of the primary crystal rotation. (**a**) 40% pulse duty cycle (**b**) 20% pulse duty cycle; and (**c**) no applied magnetic field.

Tahashi et al.^25^ determined the crystal rotation angle from XRD data using the following equation:

1 $$\theta_{{\text{F}}} = \frac{{\sum \left( {I_{{{\text{hkl}}}} \times \theta_{{{\text{hkl}}}} } \right)}}{{\sum I_{{{\text{hkl}}}} }}$$

where $\theta_{{\text{F}}}$ is the angle between the (hk0) plane and the c-axis; $\theta_{{{\text{hkl}}}}$ is the angle between the (hkl) and (00n) planes; and $I_{{{\text{hkl}}}}$ is the XRD intensity of the (hkl) plane. Equation (1) was used to obtain the rotation angle of the crystal from the XRD data (Fig. 6). Compared with the solidified specimen that was not subjected to a magnetic field, the (111) crystal plane was rotated 13° toward the magnetic field direction under the 40% pulse duty cycle but only 3° for under the 20% pulse duty cycle. Thus, the pulse duty cycle significantly impacted the rotation process.

### Crystal rotation process

**Figure 7:**
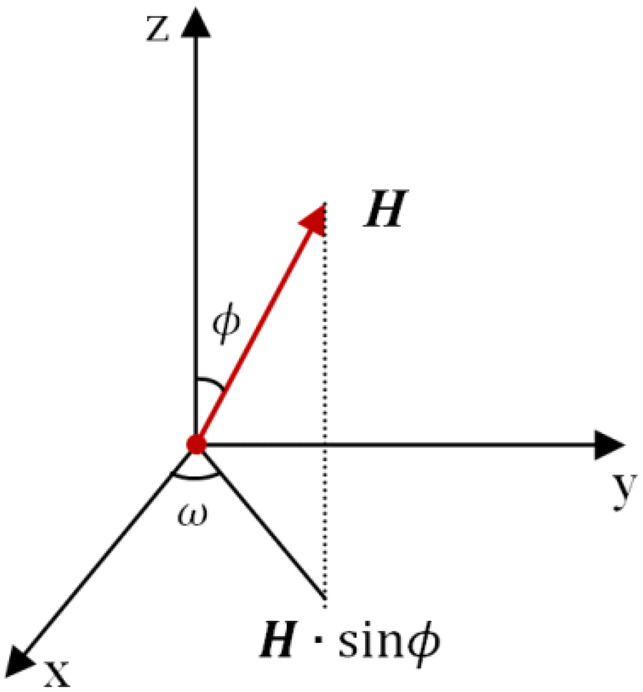
Coordinates of magnetic anisotropic crystal.

Neglecting thermal convection, the magnetic anisotropy of the crystal causes the primary nucleus to rotate under the influence of a pulsed magnetic field. The magnetic susceptibility of the Al–Zn–Mg–Cu alloy is a direction function of the magnetic field intensity, which can be expressed as $\chi = \chi \left( {\omega ,\phi } \right)$. As the nucleus grows into the α-Al crystal, rotation is driven by the magnetic dipole moment induced by the external magnetic field and the crystal magnetic anisotropy. When the magnetic field is parallel to the maximum magnetic susceptibility, the magnetic energy provides less interaction for the rotation. As shown in Fig. 7, the primary α-Al crystal was placed in an x–y orthogonal coordinate system, where the x-axis is defined as the easy magnetization axis for the magnetic susceptibility $\chi_{1}$, and the y-axis is defined as the hard magnetization axis for the magnetic susceptibility $\chi_{2}$. The projection angle between the easy magnetization axis and the magnetic field direction on the x–y plane is $\omega$. The magnetic field strength in the x–y plane is as follows:

2 $${\varvec{H}}_{{{\mathbf{x}} - {\mathbf{y}}}} = {\varvec{H}} \cdot {\sin}\phi$$

Simplifying Eq. (2) using a two-dimensional vector analysis yields Eq. (3):

3 $${\varvec{H}}_{{{\mathbf{x}} - {\mathbf{y}}}} = {\varvec{H}}_{{\mathbf{x}}} {\cos}\omega + {\varvec{H}}_{{\mathbf{y}}} {\sin}\omega$$

The total magnetization can be expressed as follows:

4 $${\varvec{M}}_{s} = {\varvec{M}}_{1} {\cos}\omega + {\varvec{M}}_{2} {\sin}\omega$$

The magnetization along the easy magnetization axis and along the hard magnetization axis can be expressed as Eqs. (5) and (6), respectively:

5 $${\varvec{M}}_{1} = \chi_{1} {\varvec{H}}_{{{\text{x}} - {\text{y}}}} {\cos}\omega$$

6 $${\varvec{M}}_{2} = \chi_{2} {\varvec{H}}_{{{\text{x}} - {\text{y}}}} {\sin}\omega$$

The α-Al crystal rotates and orients under the action of the magnetization force and reaches the equilibrium position with the smallest magnetic energy. The magnetic energy can be expressed as follows:

7 $$E_{{\text{m}}} \left( {\omega ,{\varvec{H}}} \right) = - 1/2V_{{\text{s}}} {\varvec{H}}_{{{\text{x}} - {\text{y}}}}^{2} \left[ {\chi_{2} + (\chi_{1} - \chi_{2} } \right){\cos}^{2} \omega ]$$

8 $${\text{When}}\quad \omega = 0,\quad E_{{\text{m}}} \left( {0,{\varvec{H}}} \right) = - 1/2V_{{\text{s}}} \chi_{1} {\varvec{H}}_{{{\text{x}} - {\text{y}}}}^{2}$$

9 $${\text{When}}\quad \omega = \pi /2,\quad E_{{\text{m}}} \left( {\pi /2,{\varvec{H}}} \right) = - 1/2V_{{\text{s}}} \chi_{2} {\varvec{H}}_{{{\text{x}} - {\text{y}}}}^{2}$$

where $V_{{\text{s}}}$ is the volume of a unit crystal, and the crystal rotation direction is determined by the paramagnetic susceptibility along each crystal axis. Since $\chi_{1} > \chi_{2}$, $E_{{\text{m}}} \left( {0,{\varvec{H}}} \right) < E_{{\text{m}}} \left( {\pi /2,{\varvec{H}}} \right)$. The rotation of the primary α-Al crystal in the magnetic field is affected by the inertial force, viscous force, Lorenz force and magnetization force, among which the inertial moment and magnetic moment are the main driving force. The equation of rotation motion caused by a magnetic field is expressed as Eq. (10)^26^:

10 $$\mathop {\frac{2}{5}\rho r^{5} \frac{{d^{2} \omega }}{{dt^{2} }}}\limits_{{\left( {{\text{Inertial}}\;{\text{force}}} \right)}} + \mathop {8\pi \eta r^{3} \frac{d\omega }{{dt}}}\limits_{{\left( {{\text{Viscous}}\;{\text{force}}} \right)}} + \mathop {\frac{4}{15}\pi r^{5} GB^{2} \frac{d\omega }{{dt}}}\limits_{{\left( {{\text{Lorenz}}\;{\text{force}}} \right)}} + \mathop {\frac{1}{{2\mu_{0} }}V_{{\text{s}}} B^{2} \left( {\chi_{1} - \chi_{2} } \right){\sin}2\omega }\limits_{{\left( {{\text{Magnetization}}\;{\text{force}}} \right)}} = 0$$

$G$ is the electrical conductivity, $\mu_{0}$ is the permeability of a vacuum, $\eta$ is the viscosity, and *r* is the grain radius. Assuming that the inertial force is the main driving force for crystal rotation, the ratio of the inertial force term to the sum of the viscous force term and the Lorenz force term is equal to 1:

11 $$\begin{aligned} & \frac{{{\text{Inertial}}\;{\text{ force}}\;{\text{ term}}}}{{{\text{Viscous}}\;{\text{ force}}\;{\text{ term}} + {\text{Lorenz}}\;{\text{ force}}\;{\text{ term }}}} = \frac{{\frac{2}{5}\rho r^{5} \frac{{d^{2} \theta }}{{dt^{2} }}}}{{8\pi \eta r^{3} \frac{d\theta }{{dt}} + \frac{4}{15}\pi r^{5} GB^{2} \frac{d\theta }{{dt}}}} \\ & \quad \approx \frac{{3\rho r^{2} }}{{2\pi \left( {30\eta + r^{2} GB^{2} } \right)t^{^{\prime}} }} = 1 \\ \end{aligned}$$

where $t^{^{\prime}}$ is the characteristic time. Substituting the physical parameters of Al ($\rho$ = 2,700 kg/m^3^, $\eta$ = 0.0035 kg m^−1^ s^−1^, $G$ = 3.538 × 10^7^ Ω^−1^ m^−1^) into Eq. (11), and assuming the magnetic flux density is 0.1 T, the action time of the inertial force of a grain with a radius of 10 μm is only 10^–6^ s (while for a grain radius of 1 μm the value is only 10^–8^ s), and the inertial force term can be ignored. Equation (10) can be simplified as follows:

12 $$8\pi \eta r^{3} \frac{d\omega }{{dt}} + \frac{4}{15}\pi r^{5} GB^{2} \frac{d\omega }{{dt}} + \frac{2\pi }{{3\mu_{0} }}r^{3} B^{2} \left( {\chi_{1} - \chi_{2} } \right){\sin}2\omega = 0{ }$$

Equation (13) is obtained by integrating Eq. (12):

13 $$\frac{{{\tan}\omega }}{{{\tan}\omega_{0} }} = \exp \left[ { - \frac{{5tB^{2} \left( {\chi_{1} - \chi_{2} } \right)}}{{\mu_{0} \left( {30\eta + r^{2} GB^{2} } \right)}}} \right]$$

where $\omega_{0}$ is the initial angle between the magnetic field and the easy magnetization axis, $\omega$ is the final angle after rotation, and $t$ is the crystal rotation time or the action time of the magnetic field. For intermittent pulsed electromagnetic fields that do not consider the inertial force, the action time of the magnetic field can be defined as *αt* after introducing a duty cycle of *α*. The equation for the crystal rotation under the pulsed magnetic field is obtained by Eq. (14):

14 $$\frac{{{\tan}\omega }}{{{\tan}\omega_{0} }} = \exp \left[ { - \frac{{5\alpha tB^{2} \left( {\chi_{1} - \chi_{2} } \right)}}{{\mu_{0} \left( {30\eta + r^{2} GB^{2} } \right)}}} \right]$$

**Figure 8:**
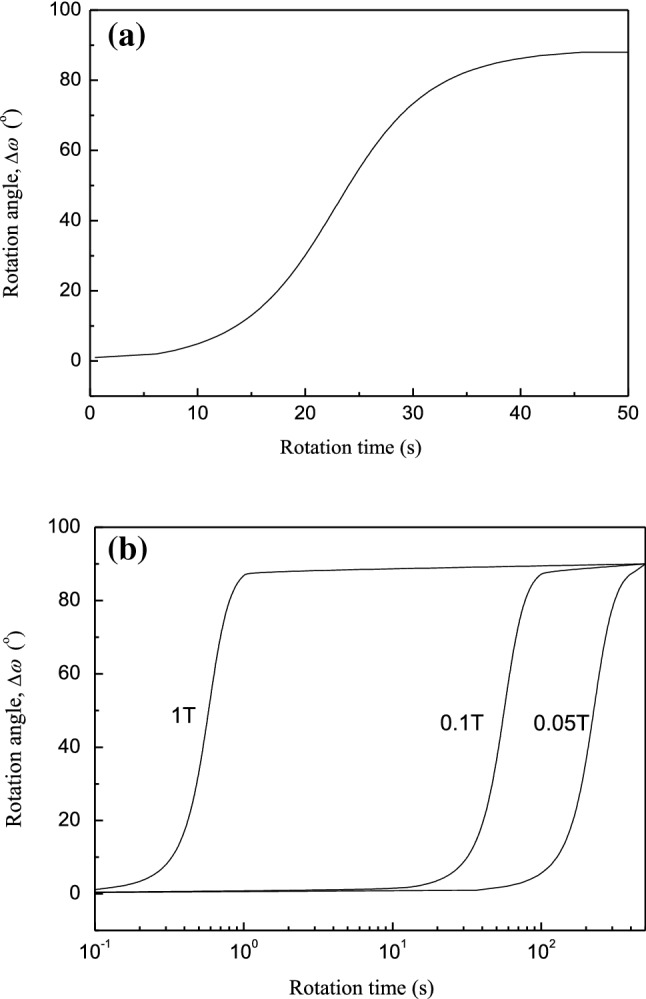
Rotation of a grain with a radius of 10 μm under a pulsed magnetic field: (**a**) rotation angle versus time for 153-mT magnetic flux density^20^ ©2017 ASM International; and (**b**) relationship between magnetic flux density and rotation time.

The difference between the magnetic susceptibility along the easy magnetization axis and the magnetic susceptibility along the hard magnetization axis is 0.5 × 10^–6^. When the pulse duty cycle is 40%, Eq. (14) is used to calculate the dependence of the rotation angle range ($\omega = \omega - \omega_{0}$) on the applied time and the magnitude of the magnetic field, which was obtained using Eq. (14) and a pulse duty cycle of 40%. Figure 8a shows the rotation of a 10-μm grain under a 153-mT pulsed magnetic field. At the grain initial position, the magnetic field was almost perpendicular to the easy magnetization axis. After 20 s, the grain rotated 31°, corresponding to 34% of the rotation process. The rotation was completed in 45 s. The grains formed with the preferred orientation. Therefore, the larger the duty cycle of the magnetic field is, the smaller the time for complete rotation of the crystal is, which is consistent with the experimental results shown in Fig. 6. Figure 8b shows the effect of the magnetic flux density on the rotation process. The crystal completed rotation within 1 s for a magnetic flux density of 1 T and 186 s for a magnetic flux density of 50 mT. The higher the magnetic flux density is, the shorter the rotation time is. Therefore, the magnetic flux density significantly influences the preferred orientation.

**Figure 9:**
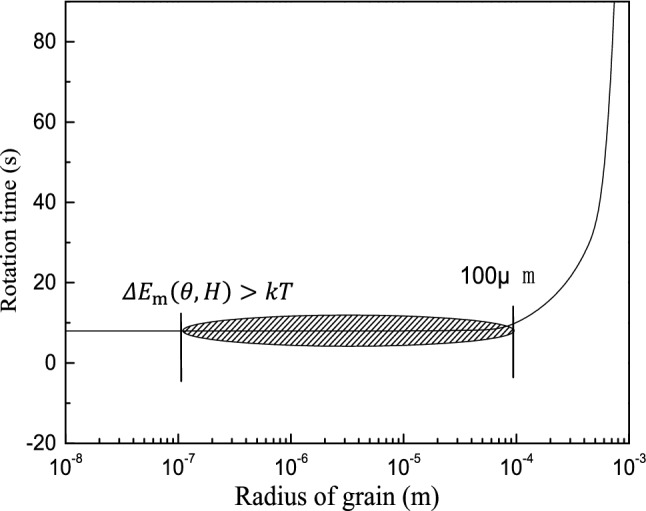
Relationship between grain size and rotation time for 153-mT magnetic flux density.

Figure 9 shows the relationship between the grain size and the rotation time when the crystal is rotated by 30°. As the grain radius increases, rotation takes longer and is more difficult. Complete grain rotation is difficult to achieve for grain sizes above 10^–4^ m. Equation (10) shows that the grain radius has the most significant effect on the Lorentz force term. The Lorentz force does not have a prominent effect for relatively small grain sizes but hinders grain rotation for grain sizes above 10^–4^ m. Thus, a small grain size is an important condition for forming the preferred orientation. The effect of the magnetic energy depends on the grain radius, the magnetic flux density, and the magnetic susceptibility. The effect of temperature on the grain orientation should be considered when the grain radius and magnetic susceptibility are temperature-dependent. The following equations hold when the magnetization energy difference is greater than thermal fluctuations:

15 $$\Delta E_{{\text{m}}} > {\text{k}}T$$

16 $$1/2V_{{\text{s}}} \chi H^{2} > {\text{k}}T$$

where k is the Boltzmann constant, and *T* is the temperature. At 650 °C, which is near the solidus temperature, the critical nucleus radius is 225 nm when the magnetization energy generated under a 0.1 T field is higher than the thermal fluctuations in the system. To rotate the crystal by 30° in a shorter time, the critical grain size of the rotation crystal should be maintained between 225 nm and 100 μm. We theoretically calculate the motion form of the primary phase under a pulsed electromagnetic field using studies in the literature^16,27^.

## Effect of pulsed electromagnetic field on crystal structure

### Effect of pulsed electromagnetic field on lattice constant

**Figure 10:**
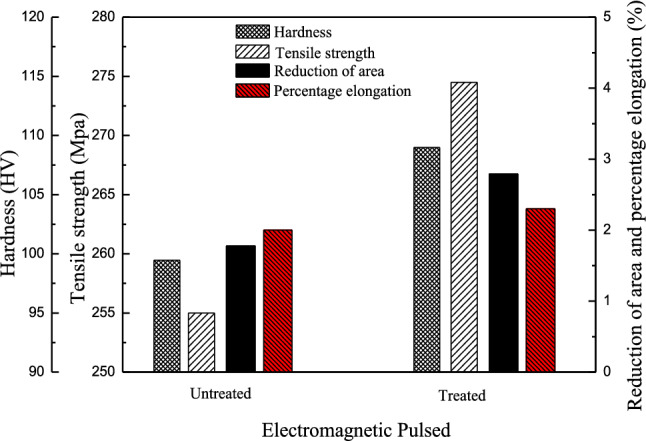
Mechanical properties of 7A04 alloy with and without electromagnetic pulse treatment.

The crystal structure and crystal lattice characteristics of α-Al affect the fatigue and plastic instability of the material. Figure 10 shows the mechanical properties of the 7A04 alloy. After electromagnetic pulse treatment with pulse duty cycles of 20%, the tensile strength of the material increased by almost 20 MPa, and the hardness and toughness were improved. Small changes in the lattice constant can often cause significant changes to the properties, structure and performance of an alloy^28^. In the molten Al–Cu–Mg–Zn alloy system, the motion and collision of solute and solvent atoms result in the formation of a stable substitutional solid solution crystal after solidification. During the formation of the condensed phase, the difference between the solute and solvent atomic radii result in local lattice distortion around the solute atoms, which creates a structural stress field. The lattice constant reflects the structural deformation induced by lattice distortion. When the radius of a solute atom is larger than that of a solvent atom, the lattice constant increases significantly; otherwise, the lattice constant decreases. The measured XRD data were substituted into the following formula to calculate the lattice constant:

17 $$a = \frac{{\lambda \sqrt {h^{2} + k^{2} + l^{2} } }}{{2{\sin}\theta }}$$

where $h, k, l$ are the Miller indices, and $\lambda$ is the X-ray wavelength. Table 2 shows that as the duty cycle of the pulsed magnetic field increases, both the lattice constant and the interplanar spacing increase, indicating that a relatively loose arrangement of atoms. This result is especially evident from the high intensities of the diffraction peaks corresponding to the (111) and (200) planes. The duty cycle is an important parameter for maintaining pulse characteristics. The fluctuation frequency of the electromagnetic field promotes atomic vibrations that facilitate the formation of vacancies and the migration of atoms. Thus, the pulsed magnetic field characteristics can affect either the frequency of atomic vibrations or the activation entropy in the atomic thermal process. Assuming that the change in the lattice constant of α-Al is caused by the magnetic field^29^,

18 $$\Delta a \equiv a_{{\text{m}}} - a_{0} = f\left( {X_{{{\text{Cu}},{\text{Mg}},{\text{Zn}}}} } \right) + f\left( {Y_{{{\text{Cu}},{\text{Mg}},{\text{Zn}}}} } \right)$$

where $a_{{\text{m}}}$ is the lattice constant of α-Al after magnetic treatment; $a_{0}$ is the lattice constant of pure Al; and $X_{{{\text{Cu}},{\text{Mg}},{\text{Zn}}}}$ is the solubility of each solute element; $f\left( {X_{{{\text{Cu}},{\text{Mg}},{\text{Zn}}}} } \right)$ is the average change in the lattice constant of α-Al caused by the dissolution of the solute atoms; $Y_{{{\text{Cu}},{\text{Mg}},{\text{Zn}}}}$ is the volume fraction of each solute element; and $f\left( {Y_{{{\text{Cu}},{\text{Mg}},{\text{Zn}}}} } \right)$ is the change in the lattice constant caused by the difference between the thermal expansion of the solutes and the matrix and depends nonlinearly on the temperature. The α-Al lattice constant during solidification is affected by the solubility and volume fraction of the solute elements and the temperature. When the last two factors remain unchanged, the solubility of the solute elements in the matrix becomes the dominant factor.

**Table 2:** Experimental α-Al crystal cell parameters.

Miller index (hkl)	State	Lattice constant, a (Å)	Interplanar spacing, d (Å)	Rate of change,${\updelta } = \frac{{{\text{a}}_{0} - {\text{a}}}}{{{\text{a}}_{0} }} \times 100$%
(111)	Untreated (a_0_)	4.0468	2.3364	–
	Treated with 20% duty ratios (a)	4.0509	2.3388	0.101315
	Treated with 40% duty ratios (a)	4.0549	2.3411	0.200158
(200)	Untreated (a_0_)	4.0480	1.0119	–
	Treated with 20% duty ratios (a)	4.0514	1.0129	0.083992
	Treated with 40% duty ratios (a)	4.0548	1.0137	0.167984
(220)	Untreated (a_0_)	4.0539	0.7166	-
	Treated with 20% duty ratios (a)	4.0505	0.7160	− 0.08387
	Treated with 40% duty ratios (a)	4.0572	0.7172	0.081403
(311)	Untreated (a_0_)	4.0500	1.2211	-
	Treated with 20% duty ratios (a)	4.0518	1.2217	0.044444
	Treated with 40% duty ratios (a)	4.0561	1.2230	0.150617

**Figure 11:**
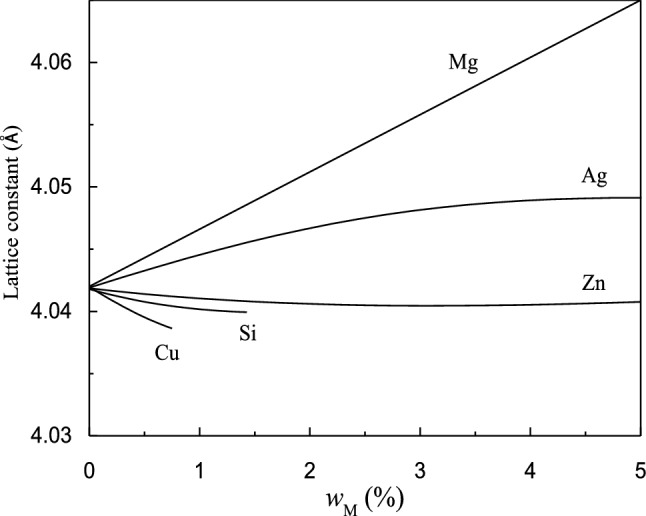
Variation in lattice constants caused by substitutional solid solution formed by solute elements in α-Al matrix.

Figure 11 shows the variation in the lattice constant induced by the dissolution of the main solute elements of the Al–Zn–Mg–Cu alloy into the matrix^30^. Generally, a 1%-increase in the Mg solubility increases the α-Al lattice constant by 0.005 Å. Similarly, a 1%-increase in the Zn solid solubility decreases the α-Al lattice constant by 0.00054 Å and decreases the lattice constant of the α-Al (Cu) solid solution^31^. Therefore, the increase in the lattice constant results from the increased solid solubility of Mg in α-Al. When an Al–Zn–Mg–Cu alloy forms a condensed phase under a pulsed electromagnetic field, the probability of nuclei collision increases and atoms exchange frequently, improving the solid solubility of the solute elements in α-Al. The main strengthening elements of the Al–Zn–Mg–Cu alloys are Zn and Mg. The main function of Cu is to improve the corrosion resistance of the matrix. Thus, pulsed electromagnetic field treatment is a novel means of enhancing the overall properties of a solid solution.

### Effect of pulsed electromagnetic field on microstrain

**Figure 12:**
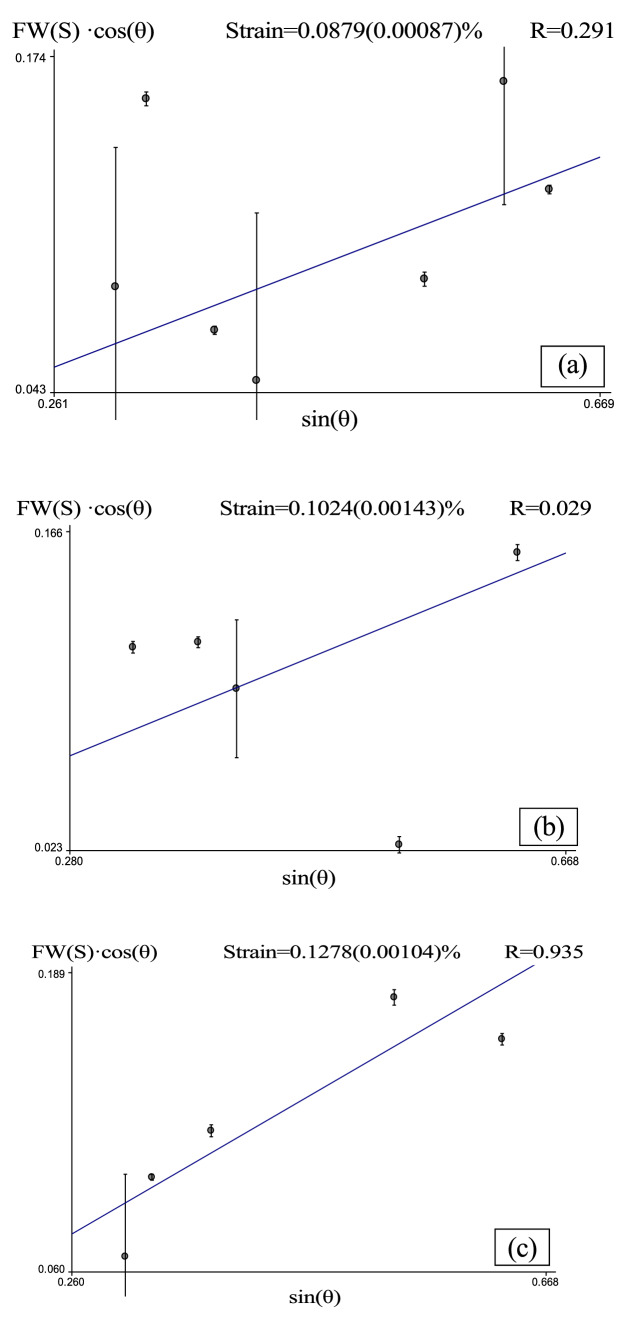
Microstrain in solidified Al–Zn–Mg–Cu aluminum alloy: (**a**) untreated and treated by 153-mT pulsed magnetic field at (**b**) 20% duty cycle and (**c**) 40% duty cycle.

An appropriate level of microstrain can strengthen the microstructure of a crystal, whereas excessively high microstrain accelerates the formation of intercrystalline microcracks^32^. During solidification, the effect of the pulsed electromagnetic field on the lattice constant induced by is reflected in the microstrain. Macherauch^33^ classified two types of microstress that occur to maintain mechanical equilibrium: stress over the scale of a few grains and the atomic-scale internal stress of a grain^34^. Microstrain broadens XRD peaks and decreases the diffraction intensity. A deconvolution method can be used to calculate the actual width increment of a diffraction peak induced by the microstrain, FW*(S*):

19 $${\text{FW}}\left( S \right)^{{\text{D}}} = {\text{ FWHM}}^{{\text{D}}} {-}{\text{FW}}\left( l \right)^{{\text{D}}}$$

where FW(*S*) is the width increment of the XRD peak induced by the microstrain, FWHM is the full width at half maximum of the XRD peak, FW(*l*) is the base width of the XRD peak caused by the instrument, and D is the deconvolution parameter. The microstrain can be expressed as the ratio of the strain (${\Delta }$ *d*) to the interplanar spacing (*d*)^35^:

20 $${\text{Strain}}\left( {\frac{\Delta d}{d}} \right) = \frac{{{\text{FW}}\left( S \right) \cdot {\cos}\left( \theta \right)}}{{4{\sin}\left( \theta \right)}}$$

where $\theta$ is the diffraction angle, and the microstrain is proportional to the FWHM. Figure 12 shows the measured microstrain of the solidified alloy structure after pulsed electromagnetic field treatment. The microstrain increases with the duty cycle. When the pulse duty cycle reaches 20%, the microstrain of α-Al increases from *ε* = 8.79 × 10^–4^ to *ε* = 1.024 × 10^–3^; and when the duty cycle reaches 40%, the microstrain of α-Al increases to *ε* = 1.0278 × 10^–3^.

Crystal imperfections following magnetic field treatment are mainly reflected by lattice distortion. The influence of the duty cycle on the microstrain can be explained as follows. (1) When a condensed phase forms, the ordered arrangement of Al atoms inside the crystal is influenced by the pulsed magnetic field. Thus, a high number of large atoms dissolve in the matrix, and atomic interactions produce lattice distortion. The resulting intragrain or intergranular stress increases to maintain mechanical equilibrium, increasing the microstrain. (2) Precipitation of the supersaturated phase can also increase the microstrain. When the solid solution phase precipitates near a grain boundary, coherence between the precipitate and α-Al is ensured by high levels of microstrain around the grain boundary. Thus, the precipitated phase acts as a strain concentrator in the matrix. (3) The application of a pulsed electromagnetic field generates a Lorentz force in the internal microcells of the system, which can also increase the microstress. Thus, a pulsed electromagnetic field induces the dissolution of a high number of large atoms in the matrix, which distorts the lattice and is the main cause of microstrain.

## Conclusion

The combined action of pulsed magnetic energy and magnetocrystalline anisotropy can change the properties and microstructure of a material, which in turn affects performance. The following conclusions can be drawn from the results and analysis of this study.

1. The combined effects of magnetocrystalline anisotropy and pulsed magnetic energy facilitate the rotation of α-Al grains with radii ranging from 225 nm to 100 μm, with the <111> axis as the preferred growth orientation. Complete grain rotation is difficult for grain sizes above 10^–4^ m.
2. As the duty cycle of the pulsed magnetic field increases, the lattice constant and interplanar spacing increase. The characteristics of the pulsed electromagnetic field significantly affect the lattice constant of the solidified structure.
3. As the duty cycle increases, the microstrain gradually increases. When the pulse duty cycle reaches 40%, the microstrain of α-Al increases from *ε* = 8.79 × 10^–4^ to *ε* = 1.0278 × 10^–3^. The application of a pulsed electromagnetic field results in the dissolution of a high number of large atoms in the matrix, which causes lattice distortion, the main cause of microstrain.
